# The effect of expertise on the creation and evaluation of visual compositions in terms of creativity and beauty

**DOI:** 10.1038/s41598-024-64494-7

**Published:** 2024-06-13

**Authors:** Yejeong Mutter, Ronald Hübner

**Affiliations:** Department of Psychology, University of Konstanz, 78464 Konstanz, Germany

**Keywords:** Psychology, Human behaviour

## Abstract

The identification of artistically creative individuals is an important matter in the fields of art, design, and psychology. One promising approach involves assessing a person's products rather than his or her personality or cognitive processes. However, the necessity of expert involvement in such evaluations is still debated. To investigate this issue, two experiments were conducted, each consisting of a production phase and an evaluation phase. In Experiment 1, participants were asked to compose a most beautiful picture, which was subsequently assessed in terms of beauty. Experiment 2 was analogous, but participants were asked to compose a most *creative* picture, which was then assessed in terms of creativity and beauty. The results revealed that expertise did not play a crucial role in the creation or evaluation of beauty. Both experts and non-experts largely agreed on what constitutes beauty. However, when it came to the production and assessment of creative pictures, experts had an advantage. They were the only group that was able to predict a person's creativity based on the evaluation of his or her product.

## Introduction

Creativity is an important human characteristic that enables the development of innovative products, ideas, and problem solutions^1^. Unfortunately, due to its complexity, the concept of creativity is difficult to define and measure. Even if we restrict our consideration to limited areas such as visual art and design, as in the present study, many difficulties remain. For instance, artists are often seen as creative because they create art. Zaidel^2^, however, cautions against associating the verb ‘to create’ with the noun ‘creativity’, because not all artistic products are creative. This problem raises various questions such as what qualities make a work of art or product creative, who is creative, and who is authorized to judge the creativity of people and products. In this study, we have tried to contribute to answering some of these questions.

For this objective, we examined the creation and evaluation of simple pictorial compositions. Moreover, concerning evaluation, we followed a practical approach. Rather than relying on specific conceptual definitions of creativity, which are usually difficult to translate into specific assessment criteria, we relied on the collective assessment of *products*. This approach allows evaluators to make their own independent product evaluations. Specifically, we applied a method similar to the Consensual Assessment Technique (CAT) proposed by Amabile^3^, because the CAT is considered most suitable for investigating creativity in domain-specific tasks^4^.

According to this technique, one group of persons creates task-specific products, and another group, usually experts, evaluates the products with respect to creativity, aesthetic appeal, and technical skill. The validity of the CAT has been demonstrated in numerous studies^4–6^, although some doubts arose about the efficacy of expert judgments, as they were sometimes cynical, biased, or inconsistent^7,8^. In the present study we therefore took expertise into account and investigated the extent to which experts differ from non-experts in the creation and identification of creative products. However, before presenting our methods and results, we provide brief introductions to relevant concepts.

### Visual aesthetics

One field where creativity plays an important role is visual aesthetics. For producing beautiful or aesthetic pictures or objects, creativity might be helpful, if not necessary. However, not all aesthetic or beautiful pictures are considered creative and vice versa. This brings us to the question: What characterizes an aesthetic picture? In the history of aesthetics, philosophers have long attempted to answer this question by proposing normative ideals of beauty. In the nineteenth century, however, Gustav Theodor Fechner (1801–1887), who defined aesthetics as the study of “the pleasing and displeasing” and named the philosophical approach “aesthetics from above”, established a complementary approach based on experimental research, which he called “aesthetics from below”^9^.

Fechner^10^ proposed that aesthetic appreciation depends on direct, stimulus driven factors as well as on associative factors. The direct factors refer to the fast perceptual processing of low-level stimulus features such as symmetry^11^, balance^12–14^, spatial composition^15^, color combination^16^, and others^17^. In the context of aesthetics this basic low-level feature processing has also been considered as “beauty-response mechanism”^18^. Its output is mostly universal. Symmetrical shapes, for example, are usually perceived as more beautiful than asymmetrical shapes^19^.

In contrast to the direct factors, associative factors depend on the viewer’s learning history, i.e., on his or her knowledge^18,20^, and cultural background^21,22^. Fechner^10^ argued that we find an orange more pleasant than an appropriately painted wooden ball because we associate an orange with a pleasant smell, a refreshing taste, being grown on a beautiful tree, in a beautiful country, and so on^23^.

More recently, these ideas have been integrated into more complex information processing models of aesthetic experience^20^. One promising class of such models are the so-called *dual-process models*, which have been introduced on a verbal level in social psychology^24^ and in a more formalized manner in research on selective attention^25^. In the area of aesthetics, Graf and Landwehr^26^ proposed an interesting dual-process model in which fluency plays an important role. It has been hypothesized that stimuli are liked more when they can fluently be processed^27^. However, Graf and Landwehr^26^ noted that sometimes even hard-to-process objects are liked. Therefore, they developed a model that combines the processing-fluency idea with the dual-process framework^28^. They assumed an early fast and automatic processing stage where processing fluency leads to positive affective feelings on a gut level.

Graf and Landwehr^26^ further assumed that a less fluent processing at the first stage elicits negative feelings, which trigger controlled processing at a second stage, where the stimulus is processed and elaborated more deeply. However, processing at stage two can also be motivated by the viewer’s “need for cognitive enrichment”, independent of the outcome of stage one. Clearly, if a viewer wants to compare the outcome of the first stage to his or her knowledge structures, he or she can initiate controlled processing to further elaborate the stimulus. If the controlled processing at stage two is easier than expected, then this results not only in positive feelings, but also in aesthetic interest, which motivates further controlled processing. Importantly, the aesthetic evaluation resulting from the controlled processing can overwrite the evaluation from early automatic processing.

Thus, according to this dual-process account, we can distinguish between early aesthetic pleasure resulting from feelings caused by fluency at the automatic-processing stage, and a later intellectual pleasure resulting from feelings caused by interest and cognitive mastering. Although these two types of feelings might lead to different aesthetic experiences, neuroscientific evidence suggests that they nevertheless have a *common currency*, i.e., a common neurophysiological origin^29^.

From these considerations it follows that beauty assessments can be based on early more perceptual as well as on late more cognitive processing, where the latter largely depends on the expertise and motivation of the viewer. For the evaluation of creativity, however, it can be assumed that it is mainly based on cognitive processes and, therefore, strongly depends on the expertise of the viewer. Aesthetic interest might be an important motivation in this context^30^. A demanding work of art, for instance, might be processed easier by experts than by non-experts, and therefore elicit aesthetic interest and further elaboration in the former but not in the latter. This difference in interest could then affect the evaluation of the creativity of the work.

### Creativity and its assessment

Already Fechner^9^ related aesthetics to creativity. In fact, one of the three methods he invented to investigate people’s aesthetic appreciation is the “method of production”, which often requires more or less creativity and is also used in the present study. After more than a century of empirical research, though, creativity has become a broad and complex field. Therefore, to provide some structure for its investigation, various frameworks have been proposed. A widely known example is the Four P’s proposed by Rhodes^31^, which define Person, Product, Process, and Press (i.e., environment) as meaningful units of research. Later, an extended framework, the Five A’s, has been introduced by Glăveanu^32^, which distinguishes Actors, Audiences, Actions, Artifacts, and Affordances. Because these frameworks are mainly concerned with how creativity is operationalized, a further framework has been proposed, the Four C’s: *mini-c* (transformative learning, personally meaningful creative insights), *little-c* (everyday creativity), *Pro-c* (professional level of creativity), and *Big-C* (eminent creativity), which is more focused on developmental steps of individuals. For an overview, see Kaufman and Glăveanu^33^.

Initially, creativity research was mainly concerned with persons or actors and the cognitive processes involved in fostering their creativity. A well-known example of such a process is *divergent thinking*, introduced by Guilford^34^. It has been assumed that the ability to think divergently, as opposed to convergently, allows for flexible exploration of multiple possible solutions to a problem. Later, Mednick^35^ emphasized the associative nature of creative thinking, arguing that the synthesis of two or more distinct concepts produces creative ideas. According to Welling^36^, these creativity promoting associations can occur in analogical, combinatorial, and abstract thinking.

Divergent thinking and associative concepts have been at the core of creativity tests. Such tests were developed in verbal and figural versions and measured the variety and originality of the responses as well as the speed of idea generation^37^. Unfortunately, these tests are not appropriate for domain-specific creativity such as artistic creation^4^. For example, although figural divergent-thinking tests consist of picture-based tasks, they only measure figural or spatial reasoning ability without considering aesthetic factors. Consequently, researchers in the area of aesthetics and art searched for alternative approaches and finally came up with the idea to measure the creativity of an artwork or product rather than the creative process^38^.

A well-known method in this respect is the Consensual Assessment Technique (CAT) developed by Amabile^3^, which is also applied in the present study. For defining the CAT, she wrote: “A product or response is creative to the extent that appropriate observers independently agree it is creative.” (p. 1001). Because this consensual definition relies on subjective criteria, it overcomes the difficulty to specify objective criteria for identifying a product as creative. However, Amabile^3^ had to assume that different degrees of creativity can reliably and validly be assessed by a group of independent evaluators. Meanwhile, several studies have shown that this assumption is indeed justified for a wide range of products^38,39^. To assess the validity of the CAT with respect to new product types, it can be helpful to have the corresponding products also evaluated on dimensions other than creativity, e.g., aesthetic appreciation^39^. This way, the independence of the creativity judgment from the other dimensions can be investigated.

If inferences about the creativity of the creators are to be drawn from differences in product creativity, then it is necessary that creative performance on the task does not depend on the practical skills of the participants. Therefore, paper collage tasks are often used as in Amabile^3^, requiring participants to create designs by gluing pieces of paper of different shapes, colors, and sizes onto a piece of cardboard. Alternatively, Tangram shapes were also used^40^. Nowadays, such designs can easily be created on a computer, as in the present study.

Because the CAT is highly flexible and can therefore be adapted to a variety of design and research situations, it has been successfully used in many studies in different fields to investigate the effects of various conditions on product creativity. Moreover, due to its similarity to creativity judgments in the real world, it is assumed that the CAT has a high degree of ecological validity^39^.

### Prototypicality and novelty

As mentioned earlier, stimuli that are processed fluently usually elicit pleasure, and are therefore often liked. An essential part of mental processing is categorization which proceeds more efficiently the more similar the stimulus is to a prototype, i.e., the most typical exemplar of a category^41^. Consequently, prototypes are usually preferred^42,43^. However, the degree of elicited pleasure also depends on the type of the category. Whitfield^30^, for instance, distinguished closed from open categories. For closed categories, which might be wired-in for specific stimuli (e.g., bodily features, colors) and therefore are related to Fechner’s^10^ direct factors (see above), pleasure depends solely on the typicality of the stimulus as described. However, there are also open categories, i.e., acquired categories that are still under construction and, therefore, open for the integration of new exemplars. According to Whitfield^30^, processing novel stimuli can also elicit pleasure, as long as they help to characterize the category more accurately and form a more appropriate prototype. This account is similar to the dual-process model^26^, which assumes that categorization of typical objects or features proceeds automatically, while the categorization of novel or atypical stimuli requires controlled processing that may need to suppress effects of typicality.

Since new objects are not automatically categorized accurately, the motivation to categorize them more accurately depends on a person's interest^30^. When people are not interested in the object, they try to minimize their mental effort and evaluate its attractiveness based on typicality. Indeed, Creusen and Snelders^44^ showed that the relative effect of novelty increases for individuals who are more interested in the corresponding object category. Therefore, the interest in an object is a necessary condition for the assessment based on distinctiveness, i.e., novelty^45^.

The mentioned ideas support the industrial design principle ‘Most Advanced Yet Acceptable’ proposed by Loewy^46^. This MAYA principle means that a successful design should find a trade-off between novelty and typicality, by trying to be as innovative as possible while maintaining the typicality of the design as much as possible. Indeed, it has been shown that this principle is successful, at least for product design^47^.

### Expertise

Because interest modulates the relative effects of typicality and novelty, Leder and Carbon^48^ suggested that people interested in aesthetics might react more positively to novelty than others, which brings us to the effect of expertise. It can be expected that experts in the fields of art and design do not only possess a corresponding differentiated mental structure of relevant categories but are also more interested in novelty and untypical objects than non-experts. Therefore, experts suppress their initial automatic affective response to the typicality of a new object and base their judgment mainly on their extensive knowledge^49,50^. One consequence of this approach is that they value visual properties that normally impede fluent processing, such as asymmetry, complexity, or abstraction, more highly than non-experts^51^. However, because the experts may have different specialized training in the arts, their criteria and preferences are also more different from each other compared to non-experts^20^. This might even go so far that their preferences are contradictory^50,52–54^, which reduces the consistency of their judgments.

Issues of reliability and validity of judgments, and how experts differ from non-experts in this respect are also important for the assessment of creativity^4,7,49,55^. In particular, in the context of CAT, there is a debate about whether or not experts are needed for evaluating product creativity. Runco and his colleagues^7,56^, for instance, have questioned whether the ratings by experts are more reliable and valid than those by non-experts. Runco, et al.^7^ even argued that experts are insensitive to the subtle levels of creativity and often biased in their judgments. In their study, expert ratings were generally lower than students’ ratings and fail to show a significant difference between the most and the least creative artworks. On the other hand, Kaufman and Baer^55^ claim that non-expert judgments are invalid unless they are consistent with expert judgment. These inconclusive statements indicate that the level of expertise required for a valid assessment of product creativity depends on various factors and differs across domains^38,39^.

Beyond the dichotomy of experts and non-experts, some researchers also considered the performance of a middle group, the so-called *amateurs,* or *quasi-experts*^49,57^. They found that the ratings by this group matched those by experts to some degree, but their tastes were more in line with those of the non-experts.

Whitfield^30^ made the interesting distinction between experts who are actively involved in the production of art and design and those who are not. Although experts generally have a well elaborated mental structure of categories, only the former are practitioners with a professional interest in creating novelty, which distinguishes them from experts, such as museum curators. Whitfield^30^ hypothesized that only experts engaged in production favor novelty over prototypicality.

### The present study

As we have seen, compositions can be created to appeal to the mental structures of ordinary people by fluently activating prototypes and thereby eliciting aesthetic feelings. However, pictures can also be composed to reflect creativity and novelty to interest and impress experts. What creators end up doing depends not only on their potential creativity, but also on their intentions and expertise. The aim of the present study was to further investigate the relationships between these and related factors. In other words, we investigated how the production and assessment of beauty and creativity depends on the level of expertise. Our main hypotheses were: 1) expertise has a greater impact on creativity than on beauty because the nature of creativity has more to do with one's knowledge, and 2) experts conceptualize creativity differently and exhibit higher creativity than non-experts.

We conducted two experiments, each consisting of Part A (production) and Part B (assessment). In Part A of Experiment 1, each participant had to compose a most *beautiful* picture with a given set of simple elements, which was later evaluated in Part B. Experiment 2 was similar, except that the task was to compose a most creative picture. The two production tasks were announced as a beauty and creativity contest, respectively, and the participants were informed that a similar group of persons will evaluate their picture.

To be successful in such contests, creators must be able to abstract from their own preferences and take the perspective of the evaluators (target group). This kind of perspective-taking is one of the standard tasks of designers who create products for a specific consumer group. Experts who have a superior experience and knowledge to identify the creativity and beauty of a product^49^ might be superior in predicting what other target groups consider as beautiful and creative. But even ordinary people are able to predict the preferences of others, at least with respect to beauty, as was recently shown by Miller and Hübner^58,59^.

Following the principles of CAT^3^, participants were allowed to interpret beauty and creativity according to their own understanding with no predetermined standard, and assessments were conducted independently. However, whereas the original CAT measures aesthetic appeal, technical skill, and creativity, our assessment omitted technical skill because we asked for a digital composition, which did not require such skill.

Furthermore, different from preceding CAT research, we did not assume that the validity of judgments can be ensured by a high interrater reliability within expert or non-expert groups^60,61^. Although reliability is necessary for valid judgments, it is not sufficient, i.e., significant interrater reliability can occur despite invalid results^55^.

Finally, since Amabile^3^, some studies varied the expertise of the evaluators, but not that of the producers. Often, they were undergraduate students or children. Consequently, similar judgments between experts and non-experts could have resulted from the lay-level-only production. In our study, we therefore also varied the expertise of the producers. Unlike former research in which experts were identified based on their professional background^8,62,63^, or using a questionnaire^64,65^, we asked our participants to self-assess their level of expertise. We assumed that the self-assessment reflects their confidence in knowledge as well as task performance.

## Experiment 1A

The first part of this experiment was announced as beauty contest. That is, the participants were instructed to create the most beautiful picture from a given set of geometric elements. They were informed that the pictures of all participants would later be judged by other persons, and that the creators of the 5 best rated pictures would receive a prize of €10 each.

We expected most non-experts to create pictures that they like, i.e., compositions that are typical for the given set of elements. Such compositions should also be easier to create than atypical ones. However, even experts should create typical compositions, even if they are not their favorites, because they know that most people like such pictures^66^.

### Method

#### Participants

The 89 participants (16 men, *M*_age_ = 23.8, *SD* = 4.71) in this online experiment were students from the University of Konstanz and from the design study program at the University of Applied Sciences (HTWG) in Konstanz. We hoped that the latter group would include many experts. At the end of the study, they reported their expertise level on a four-point ordinal scale by answering the question “How much experience do you have in Arts, Design, and Visual Communication?”. Possible answers were: 1: “Almost none”, 2: “very little”, 3: “quite some”, and 4: “I am an artist/designer or the like”. The score was expected to reflect how each participant conceives their professionality. Even though HTWG students were in Master and Bachelor of Art, some declared their expertise level as low as 2. On the other hand, some non-art students at the University of Konstanz marked their expertise level as high as 3. For convenience, the expertise levels of creators from 1 to 4 will be labeled as C1, C2, C3, and C4, respectively. We consider Groups C1 and C2 as non-experts, Group C3 as amateurs or quasi-experts, and Group C4 as experts. The number of participants for each level was C1: 17 (19.1%), C2: 26 (29.2%), C3: 10 (11.2%), and C4: 36 (40.4%), respectively.

#### Procedure

The experiment was announced as a design contest. The challenge was to create the most *beautiful* pictorial composition from a given set of seven visual elements. A voucher worth €10 was promised as a reward for the best five pictures, which were determined in Experiment 1B. The computer program for the online-experiment was written in Javascript. At the beginning participants were shortly introduced to the topic and procedure of the study. After consent and providing personal information (gender, age), a specific instruction for the study’s task was presented. To achieve standardized visual quality of stimuli presentation, participants were informed that they had to use a computer. The program stopped if a mobile device was used, otherwise it switched to full-screen mode for an even perspective.

The visual elements consisted of differently sized black discs located in a 500 × 500 pixel white canvas on a medium grey background. At presentation, the discs were arranged horizontally in the center, either in increasing (the smallest on the left, the largest on the right) or decreasing order (the largest on the left, the smallest on the right). Participants were asked to move each disc at least once and finish the composition when satisfied. The discs could be moved via drag-and-drop to any point within the canvas but were not allowed to overlap.

This study was approved by the Institutional Review Board of the University of Konstanz, Germany (approval #IRB23KN011-02w) and performed in compliance with the ethical standards of the 1964 Declaration of Helsinki and its later amendments (World Medical Association, 2013) and with the ethics and safety guidelines of the University of Konstanz. Participants were informed of their right to abstain from participation in the study or to withdraw consent to participate at any time without reprisal. Their informed consent was obtained by check-marking a box before the actual study started.

### Results and discussion

#### Picture categorization

**Figure 1:**
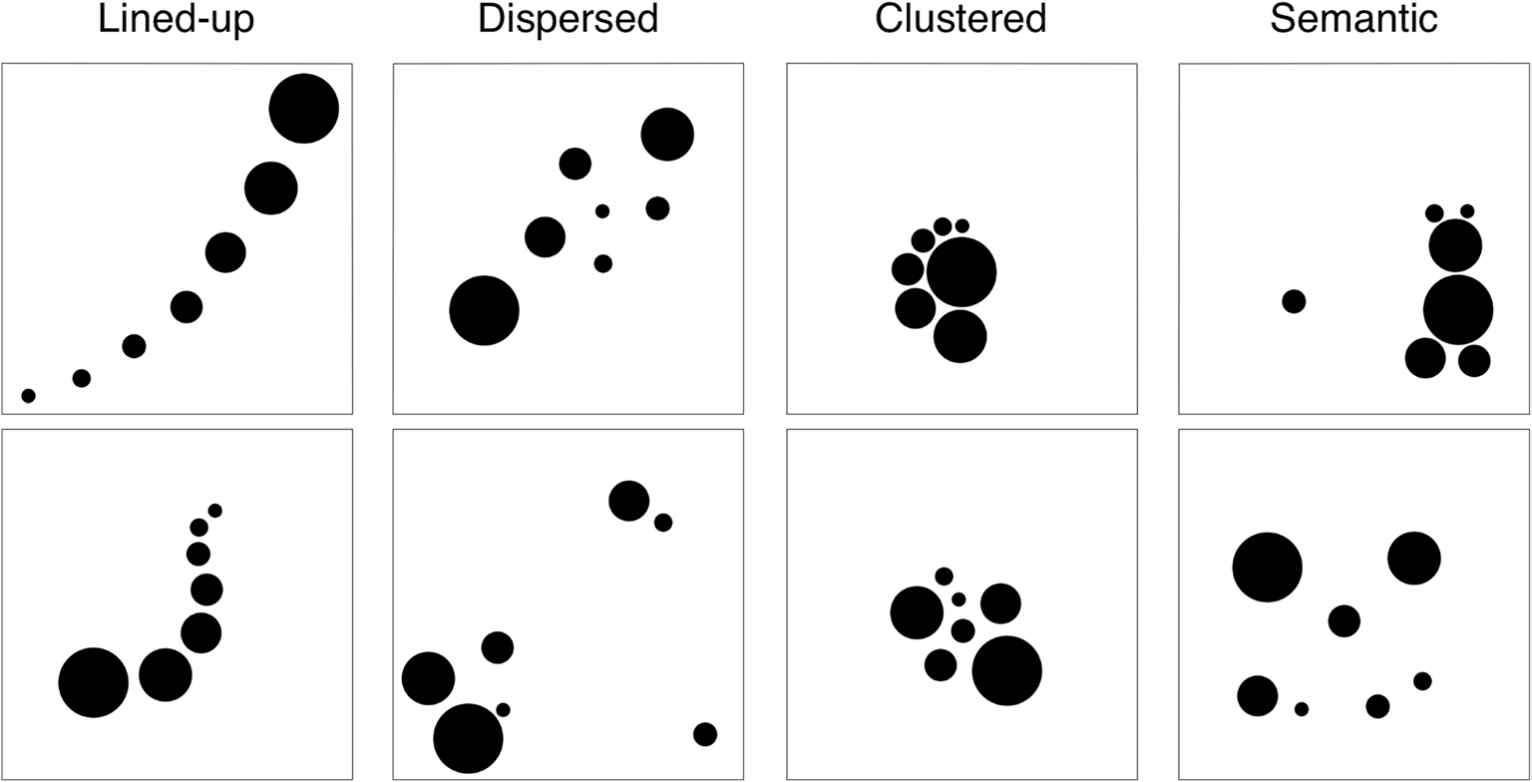
Exemplars of the four picture types created in Experiment 1A.

As a first step, the resulting 89 compositions were categorized into four picture types according to how the elements were arranged (for example pictures see Fig. 1). Compositions were categorized as *lined-up* elements type if their elements were arranged in one straight, curved, or angled line with a gradual size variation. *Dispersed* elements type compositions consist of irregularly and widely scattered elements, whereas in *clustered* elements type compositions, the elements are closely arranged as one group. Finally, if a composition can semantically be interpreted, it is categorized as *semantic*. The categorization of the 89 compositions was confirmed by five experts who achieved higher education at the Royal College of Art in London (RCA) and have worked professionally for three to ten years. However, it should be noted that many compositions could also have been categorized differently, especially those that have multiple relevant features. For example, some of the semantic compositions are also clustered. Moreover, most of the lined-up pictures can also be interpreted semantically. Thus, our categorization is mainly based on the most salient feature.

**Figure 2:**
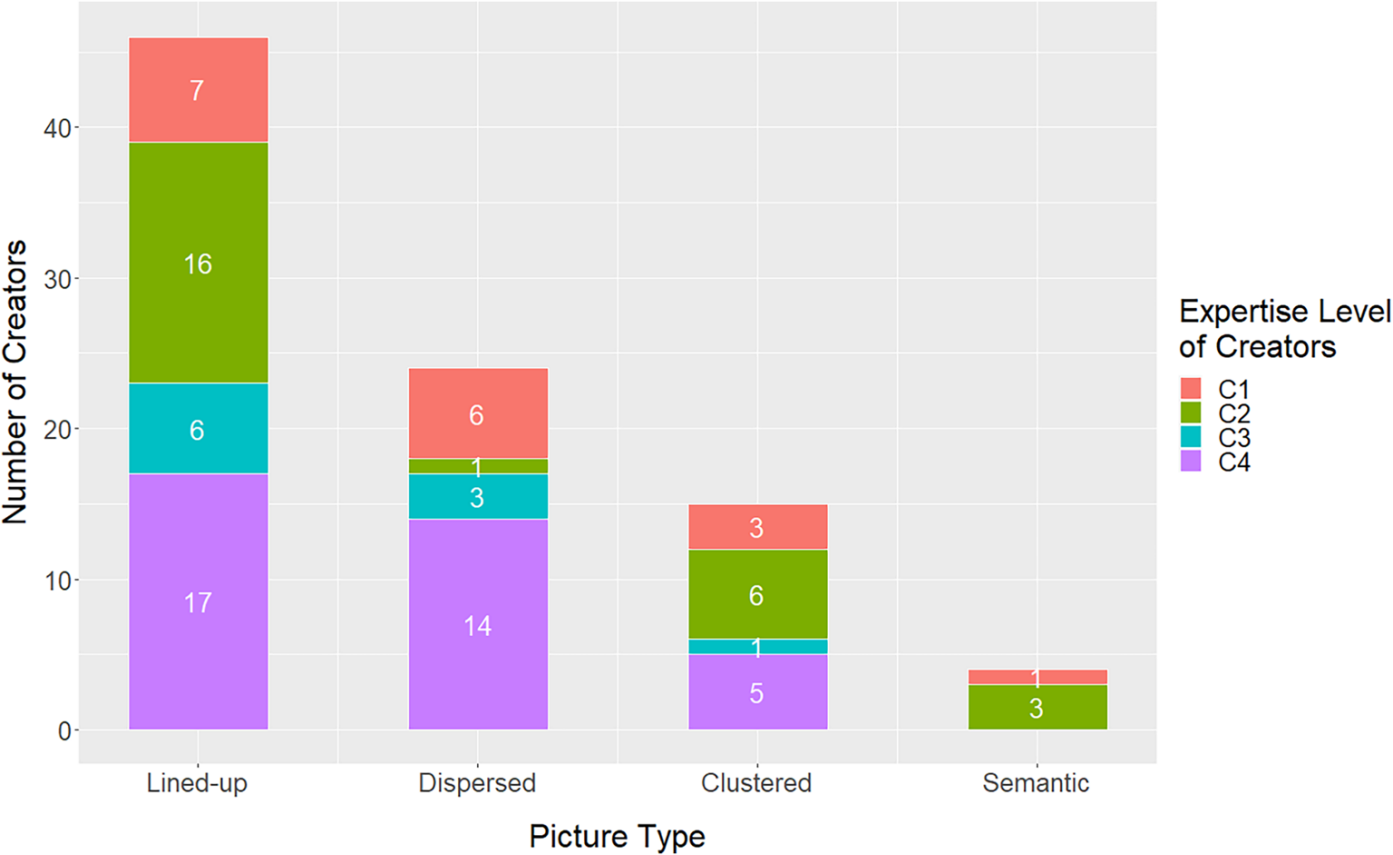
Histogram of creators' distribution among picture types.

The number of pictures per category was 46 (51.7%) for lined-up, 24 (27.0%) for dispersed, 15 (16.9%) for clustered, and 4 (4.49%) for semantic (see Fig. 2). Obviously, the most frequent pictures were those with lined up elements. If we examine the distributions taking into account the expertise level of creators, it is remarkable that more than half of the compositions of the dispersed type was created by experts (C4). In contrast, amateurs (C3) and experts (C4) did not produce semantic compositions.

#### Typicality

To investigate the role of typicality, we asked three aesthetic experts to rate whether or not each of the 89 pictures were prototypical. For assessing rater consistency, we computed the intraclass correlation coefficient (ICC)^67^ with the corresponding function in the R package IRR^68^, where we used the two-way model measuring consistency of average score^69^. As a result, 52% (46 out of 89) of the pictures were categorized as typical, with high rater consistency, ICC(C,3) = 0.795, *p* < 0.001. Importantly, as expected, 39 out of the 46 pictures (85%) with lined-up elements were judged as typical, whereas only a minority of the pictures of the other three types was judged as typical (4%, 33%, and 25%, respectively).

## Experiment 1B

In the second part of Experiment 1, the pictures created in the first part were evaluated in terms of beauty. As we have seen in Part A, in most of these pictures the elements were lined up. Moreover, the majority of these lined-up type pictures can be considered typical. Compositions that are composed of lined up elements have important features that contribute to typicality. For instance, the regular and sequential arrangement evoke the Gestalt principle of good continuation^70^ that enables the perception of a single grouped form. Consequently, such compositions facilitate rapid comprehension by viewers, and this fluent processing can be expected to influence universal aesthetic judgments in a positive manner. Interestingly, even many experts created such pictures, probably deliberately and irrespective of their personal preferences, because they presumably knew that most people find prototypical images aesthetically pleasing. In Experiment 1B, through the aesthetic evaluation task, we aimed to investigate whether evaluations differed depending on the rater’s level of expertise, along with examining the influence of aesthetic attributes such as typicality. However, we did not have a clear hypothesis about whether experts would also rate lined-up type pictures of others as beautiful.

### Method

#### Participants

The 54 participants (13 men, *M*_age_ = 26.6, *SD* = 8.76) in this online experiment were students from the University of Konstanz and from the design study program at the HTWG. Thirty-six of the participants had also participated in Part A. For completing the study, participants received a voucher worth € 3. They reported their expertise level from 1 to 4 in the same way as in Part A after the rating tasks. To distinguish the expertise levels of the raters from those of the creators, the four levels are labeled R1, R2, R3, and R4, respectively. The number of participants in each group was R1: 15 (27.8%), R2: 14 (25.9%), R3: 8 (14.8%), and R4: 17 (31.5%).

#### Stimuli and procedure

The 89 pictures composed in Experiment 1A served as stimuli. The procedure was also similar to Part A, except that, instead of creating a picture, the participants had to rate each of the randomly presented pictures according to how much they liked them on a visual analog scale that internally ranged from 1 (“I do not find it beautiful”) to 100 (“I find it beautiful”). The pictures had an extension of 500 × 500 pixel and were centered on a medium grey background. The study complied with the same ethical standards as in Experiment 1A.

### Results

#### Rater consistency

In the first step, we analyzed the consistency within and between the rater groups. As measure for the within-group and the between-group consistency, we computed the intraclass correlation coefficient and Pearson’s correlation coefficient, respectively. The results can be seen in Table 1. The ICCs show that the consistency within Group R1 and Group R2 was excellent, whereas that within Group R3 and Group R4 was good^71^. Regarding the consistency between groups, it can be seen that it decreases for Group R4 with the expertise distance to the other groups.

**Table 1:** Down-left: Interclass correlations of the mean beauty ratings. On the diagonal are the intraclass correlations (ICCs).

	R1	R2	R3	R4
R1	0.797***			
R2	0.829***	0.842***		
R3	0.812***	0.851***	0.711***	
R4	0.487***	0.623***	0.686***	0.689***

***p < 0.001.

In order to test whether the creation of a picture in Part A had an influence on the ratings, we selected the 18 people from the creator-and-rater group, which led to a distribution of expertise as similar as possible to that of the 18 participants in the rater-only group. The correlation between the mean ratings of the two groups is *r* = 0.863 (*p* < 0.001), which indicates that the creation of a picture in Part A had no substantial effect on the beauty ratings.

#### Mean ratings and winning pictures

**Figure 3:**
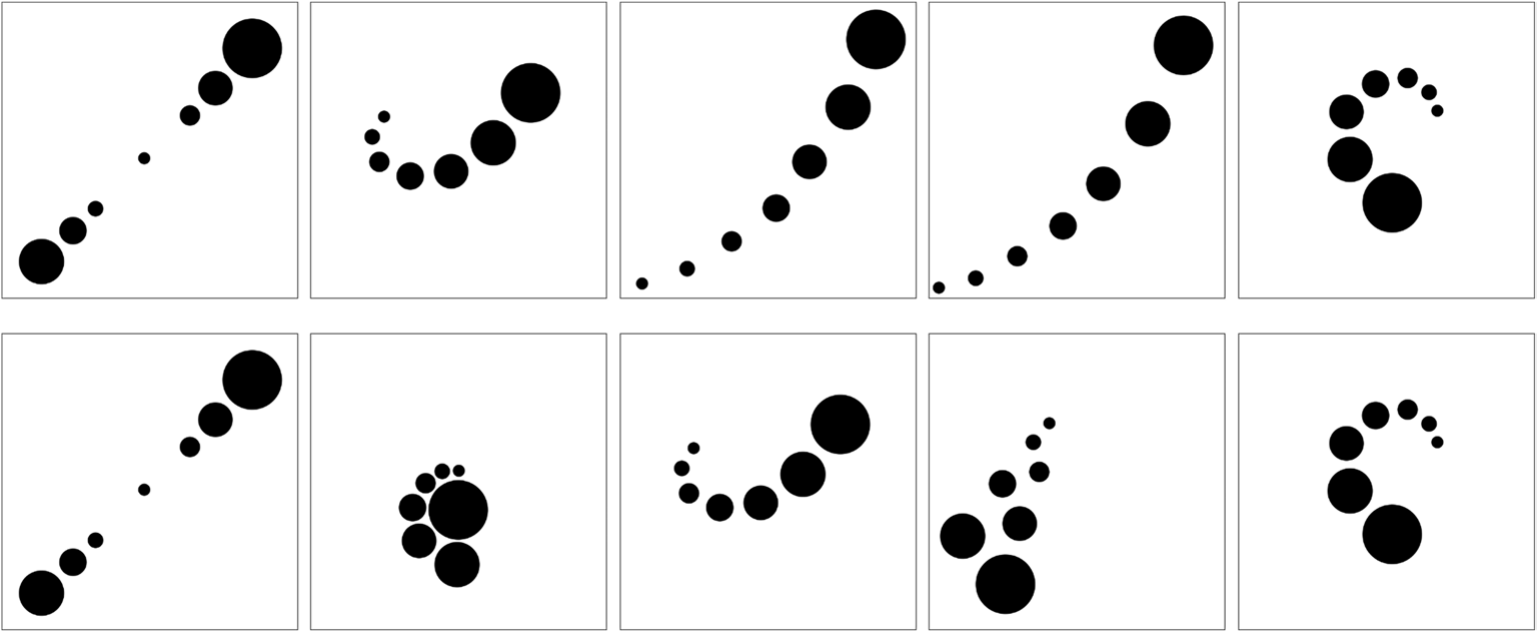
Top row: The five highest rated pictures. Mean ratings are from left to right: 64.7, 63.9, 62.1, 62.0, and 60.3. All are of lined-up type. Expertise of the creators was C2, C4, C2, C1, and C3, respectively. Bottom row: The five highest rated pictures by rater Group R4. The corresponding mean ratings are: 67.8, 63.6, 59.4, 59.3, and 56.1. The picture types are: lined-up, clustered, lined-up, lined-up, and lined-up, respectively. The expertise of the creators was: C2, C2, C4, C4, and C3.

The average beauty rating across all pictures was 42.6 (*SD* = 10.6), with mean ratings for individual pictures ranging from 19.7 to 64.7. As can be seen in Fig. 3, the five winning pictures were all the typical lined-up type. For comparison, we also added the favorite pictures of the expert group (R4) to Fig. 3. They are also of the lined-up type, with one exception.

A further result is that typical pictures, regardless of their type, were rated as more beautiful (*M* = 50.6, *SD* = 12) than atypical ones (*M* = 35.1, *SD* = 8.58), *F*(1, 88) = 80.1, *p* < 0.001.

#### Effects of picture type and expertise

**Figure 4:**
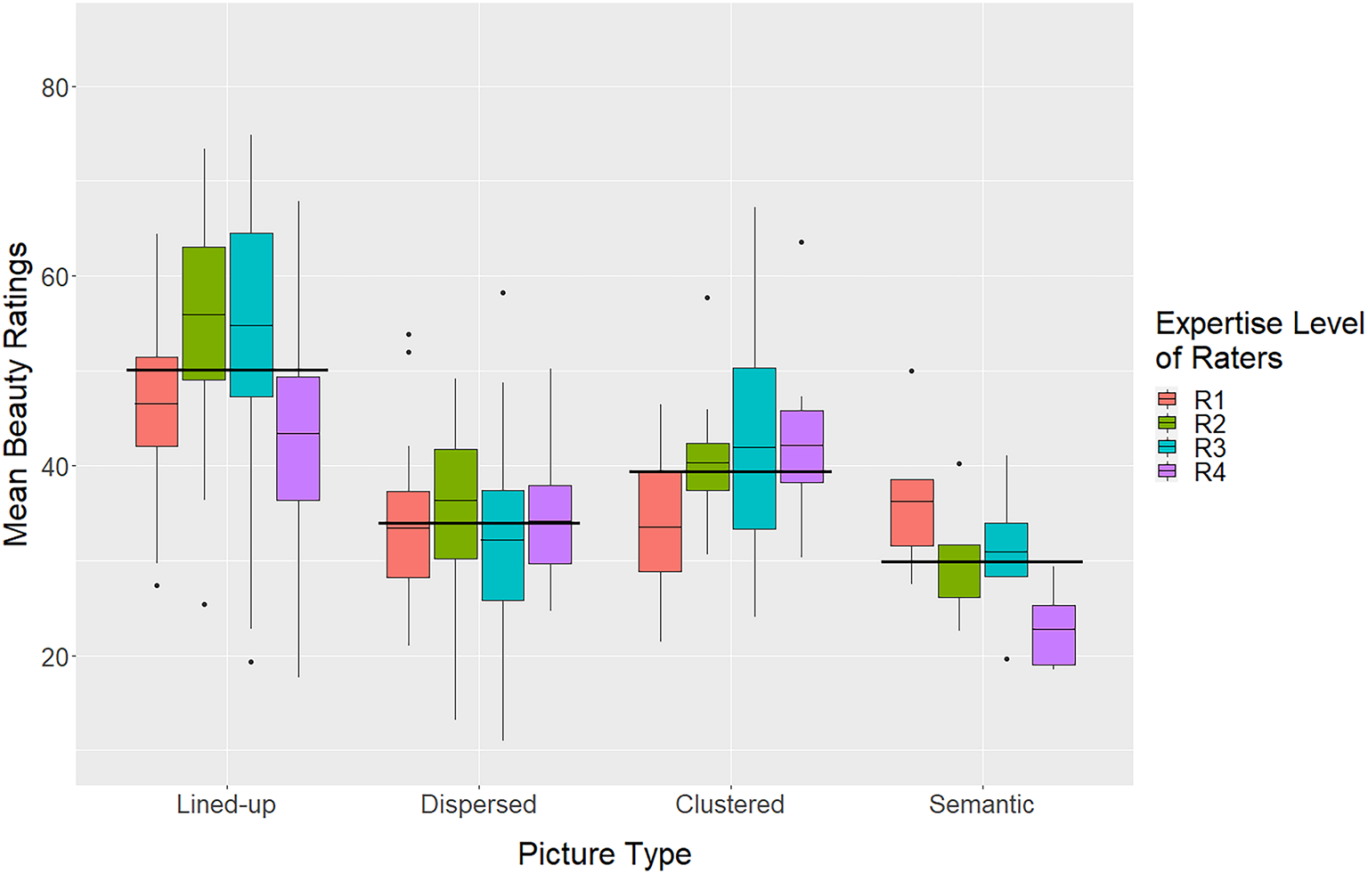
Mean beauty ratings of the different picture types (long crossbars) and corresponding mean expertise levels of the rater groups.

Figure 4 shows how the mean beauty ratings depend on the picture type and the expertise of the raters. The data were subjected to a corresponding two-way analysis of variance (ANOVA) with the within-participant factor *picture type* (lined-up, dispersed, clustered, and semantic) and the between-participants factor (rater) *expertise* (R1, R2, R3, and R4). For further analyses the Tukey HSD (honestly significant differences) test was used.

The ANOVA revealed that *picture type* had a significant main effect,* F*(3, 355) = 70.8, *p* < 0.001, PES = 0.384 (lined-up: 50.1, dispersed: 34.0, clustered: 39.5, and semantic: 30.0). Subsequent tests indicated that the lined-up pictures were rated, on average, more beautiful than those of all other types (all *p* < 0.001), and that the pictures of the clustered type were rated more beautiful than those of the dispersed type and semantic type (*p* < 0.01 for both). *Expertise* also had a significant main effect on the beauty ratings, *F*(3, 355) = 11.7, *p* < 0.001, PES 0.094. Group R2 and Group R3 rated the pictures significantly more beautiful (46.8, 45.4) than Group R1 and Group R4 (40.3, 39.8). However, there was also a significant interaction between *picture type* and *expertise*, *F*(9, 355) = 3.60, *p* < 0.001, PES = 0.087. It indicates that for the lined-up pictures the mean ratings by the rater groups R2 and R3 were significantly (*p* < 0.01) higher compared to those by groups R1 and R4 (R1: 46.5, R2: 55.9, R3: 54.7, R4:43.4), but not for the other three picture types. Thus, the significant main effect of *expertise* was largely driven by the ratings of the lined-up type pictures.

#### Predictive potential of the evaluators

An interesting question was how well the creators' expertise can be predicted by the beauty ratings of their compositions. Because expertise was scaled ordinally, we used the Spearman’s rank correlation *r*_*s*_ to calculate correlations between mean ratings and level of expertise. A corresponding overall correlation was not significant, *r*_*s*_ =  − 0.008 (*p* = 0.448). We also found no significant correlation when we looked at the individual rater groups. The correlations ranged from − 0.157 for Group R2 to 0.055 for Group R4 (*p* > 0.141 for all).

### Discussion

In the second part of our first experiment, participants with different levels of expertise rated the pictures composed in Part A in terms of their beauty. These ratings allowed us to complete the beauty contest. It turned out that all five winning pictures, i.e., those rated most beautiful in Part B, are pictures with lined up elements. This even holds for the expert (R4) ratings, with one exception. Furthermore, the pictures in this category were rated the most beautiful overall, which is no surprise, given that most of them can be considered as typical for pictures composed of the given elements.

However, the ratings of the pictures with lined up elements varied with the expertise of the raters. Although four of the five favorite pictures of the experts were also of the lined-up type, on average, Group R4 rated these pictures lower relative to other groups. However, this was not the only group. Group R1 gave similar low ratings. Thus, raters with some expertise (R2, R3) rated the images highest. Nevertheless, the mean ratings of the other picture types were generally lower, irrespective to expertise, and also did not differ significantly from each other.

The fact that only a few pictures with semantic content were composed in Part A and none of them by experts (C3, C4) seems to indicate that pictures of this type have a low aesthetic appreciation. This is supported by their numerically lower beauty ratings, especially by Group R4. Although these differences are not statistically significant, probably due to the small number of semantic pictures, they are compatible with other observations^49^.

If we consider the expertise of the creators, then it is surprising that only one of the most beautiful (winning) pictures was produced by an expert (C4), showing that the beauty of the pictures was unrelated to the expertise level of the creators. This could also explain why creator expertise cannot be predicted by the overall mean beauty ratings nor by those of individual rater groups.

Thus, by and large, the results of our first experiment are consistent with previous results. They show that typicality plays an important role for the creation and aesthetic appreciation of pictorial compositions. It seems to come quickly to mind, and therefore to be typical, to line up elements with gradual differences in size. Many participants then utilized the positive relation between typicality and beauty, presumably more or less intuitively. On the viewer’s side, such typical pictures can be easily perceived and processed, which increases their perceived beauty^27,72^. Interestingly, although expertise had some effect, it was unsystematic and relatively small. This can change when we move from beauty to creativity.

## Experiment 2A

In Experiment 1, we investigated what types of pictures are created when participants are motivated to compose a most beautiful picture with a given set of elements. As a result, many pictures with lined-up elements were produced, which were also liked most. Moreover, there was little difference between the performance of experts and non-experts in both the creation and evaluation of beauty. This might be different when the task is to compose a most *creative* picture. In this case novelty and interestingness are more important than beauty, and it is likely that experts have an advantage in this respect, compared to non-experts^49,50,63^.

Therefore, the aim of our second experiment was to investigate the production and assessment of creative pictures. For this objective, we conducted an experiment similar to Experiment 1, except that the participants were motivated to compose a most *creative* picture rather than a most beautiful one. Given the nature of creativity, as considered in the Introduction, we expected that typicality should be reduced in creative compositions, therefore, pictures with lined up elements to be less frequent. Moreover, creativity should not only be expressed by an arbitrary deviation from typicality, but the deviation should also be interesting in some sense. We hypothesized that, due to their knowledge, the creation and evaluation of such pictures should differ between experts and non-experts.

### Method

#### Participants

The 58 participants (15 men, *M*_age_ = 24.3, *SD* = 2.67) in this online experiment were students from the University of Konstanz, from the design study program at the HTWG, and from the RCA. All participants in Experiment 2 were independent of those in Experiment 1. The participants’ expertise level was registered in the same way as in the previous experiments. Given the potential for differences in expertise levels across institutions, it is important to note that there may exist some variation even among participants who self-reported their level of expertise as 4.

The data of three participants were excluded because they did not report their expertise level. Similar to Experiment 1A, the four expertise levels of the creators will be labeled C1, C2, C3, and C4, respectively. The number of participants for each level was C1: 24 (43.6%), C2: 17 (30.9%), C3: 4 (7.27%), and C4: 10 (18.2%), respectively.

#### Procedure

The procedure was also similar to that in Experiment 1A, except that the experiment was announced as a creativity contest, i.e., the task was to compose a most *creative* picture. A voucher worth €10 was offered as a prize for each of the creators of the five most creative compositions, which were determined in Experiment 2B. The experiment complied with the same ethical standards as the previous studies.

### Results and discussion

**Figure 5:**
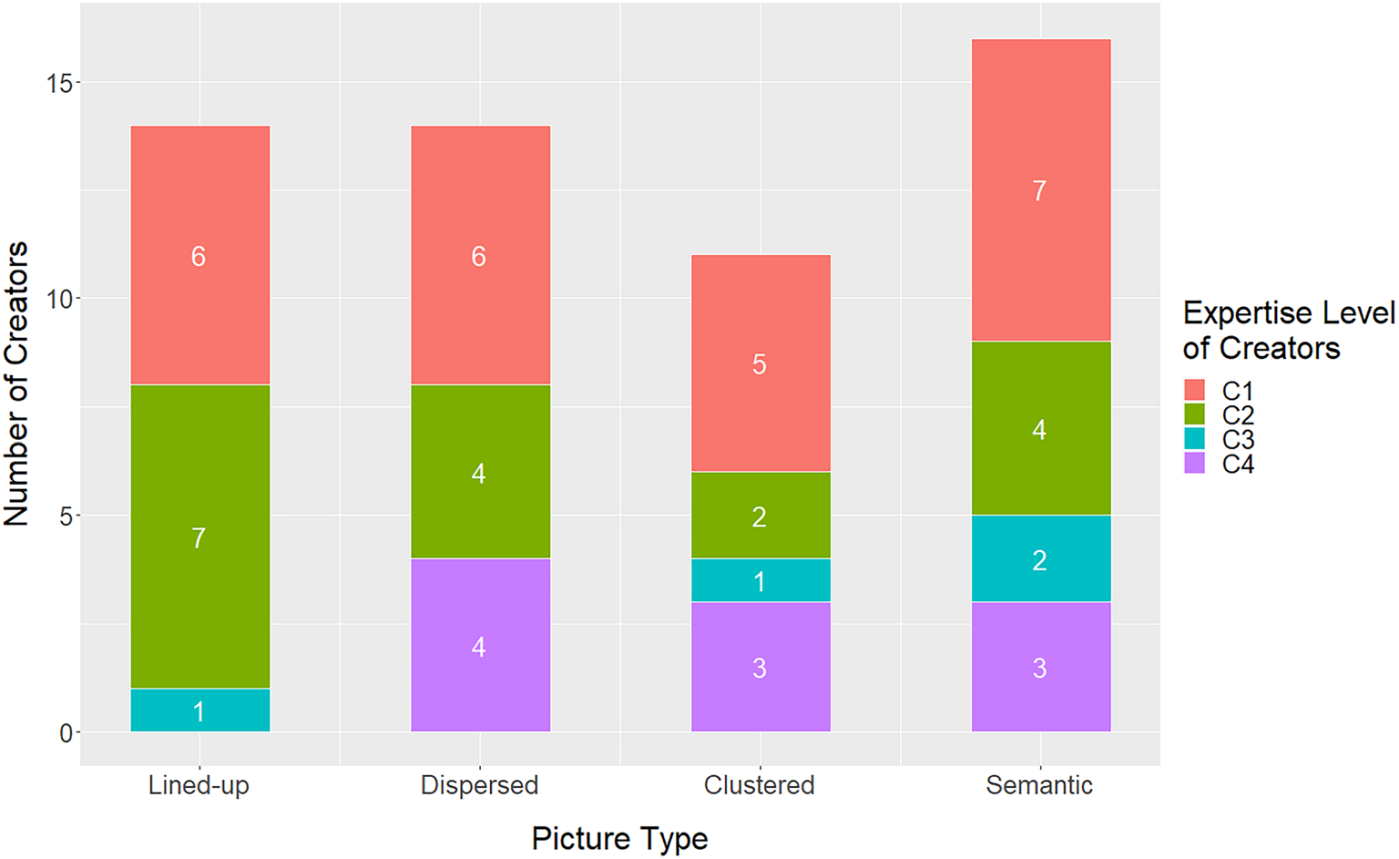
The distribution of the creators’ expertise across picture types.

All pictures were classified into the same four categories as in Experiment 1A. The categorization was confirmed by five experts of RCA graduates, independent from Experiment 1A. Of the pictures, 14 (25.5%) had lined up elements, 14 (25.5%) dispersed elements, 11 (25.0%) clustered elements, and 16 (29.0%) a semantic content. These proportions (Fig. 5) are significantly different, *Χ*^2^(3) = 20.6, *p* < 0.001 from those in Experiment 1A (Fig. 2). Obviously, the proportions in the different categories are now much more evenly distributed.

Importantly, as expected, pictures with lined up elements were produced less frequently than in Experiment 1A (26% versus 52%), *Χ*^2^(1) = 8.57, *p* = 0.003. In Experiment 1A, we could not rule out the possibility that the initial lined-up order of the elements on the display has led people to compose a similar picture. The results of Experiment 2A, do not support this concern. Interestingly, this time none of the experts (C4) composed a picture with lined up elements.

While the proportion of lined-up pictures decreased, that of the semantic type increased from 4.5 to 29%, *Χ*^2^(1) = 15.2, *p* < 0.001. This indicates that the creativity contest led to a different strategy, compared to the beauty contest in Experiment 1A. Clearly, because pictures with lined up elements are typical, they are hardly suitable for demonstrating one’s creativity. Therefore, some participants obviously assumed that creativity can better be demonstrated with a novel semantic composition.

However, it should also be noted that the distribution of expertise was somewhat different from that in Experiment 1A. Here, we had more non-experts (C1: 44% versus 19%) and fewer experts (C4: 18% versus 40%).

## Experiment 2B

In the second part of this experiment, we used the 55 pictures composed in Part A as stimuli and asked a different group of participants to rate them in terms of their creativity. The aim was to examine how creativity is evaluated by the different expert groups, and to what extent the ratings differ from the beauty assessments in Experiment 1B. In addition to creativity, the participants also had to rate the beauty of the pictures. This assessment served two purposes. First, participants should be made aware of the difference between creativity and beauty to prevent them from evaluating beauty simply as a heuristic for evaluating creativity. Second, we wanted to compare the beauty ratings for the different picture types with those in Experiment 1. This should give us insight into the effects of the task goal on the beauty ratings of the composed pictures. Because there were fewer pictures with lined up elements and more of the semantic type, compared to Experiment 1, we expected that the current pictures would be rated as less beautiful on average.

With respect to expertise, we hypothesized that experts perform better than non-experts, at least in the assessment of creativity. That is, experts should be more successful in identifying pictures created by experts.

### Method

#### Participants

The 50 participants (15 men, *M*_age_ = 26.3, *SD* = 4.33) in this online experiment were students from the University of Konstanz, from the design study program at the HTWG, and from the RCA. They did not take part in Experiment 2A and reported their expertise level in the same way as in the previous experiments. The number of participants in each group was R1: 16 (32%), R2: 13 (26%), R3: 9 (18%), and R4: 12 (24%), respectively.

#### Stimuli and procedure

As stimuli served the 55 pictures composed in Experiment 2A. The participants had to rate the randomly presented pictures in terms of creativity and beauty, respectively. For their participation each rater received a voucher worth €2. The procedure was identical to that of Experiment 1B, except that two visual analogue scales (1–100) were located below the picture, one for creativity assessment and the other for beauty assessment. The creativity scale ranged from ‘not creative’ to ‘very creative’, while the beauty scale spanned from ‘not beautiful’ to ‘very beautiful’. The experiment complied with the same ethical standards as the previous experiments.

### Results

#### Rater consistency

As a first step, we analyzed the consistency within and between the rater groups in the same way as in Experiment 1B. The values in Table 2 outside the diagonal represent the interclass correlations of the creativity and beauty ratings. As can be seen, they were highest between the groups R1 and R2. They were somewhat smaller between these groups and Group R3. Interestingly, the ratings by Group R4 did not correlate with those of groups R1 and R2 and only poorly with those of Group R3.

**Table 2:** Down-left: Interclass correlations of the beauty ratings. Up-right: Interclass correlations of the creativity ratings. On the diagonal are the corresponding intraclass correlation coefficients (ICCs).

	R1	R2	R3	R4
R1	0.804***/0.57***	0.723***	0.547***	− 0.168 (*p* = 0.22)
R2	0.886***	0.792***/0.752***	0.584***	− 0.147 (*p* = 0.28)
R3	0.588***	0.619***	0.516***/0.483***	0.28* (*p* = 0.04)
R4	− 0.096 (*p* = 0.48)	− 0.066 (*p* = 0.63)	0.082 (*p* = 0.55)	0.038 (*p* = 0.40)/0.485***

***p < 0.001, *p < 0.05.

The ICCs are shown on the diagonal of Table 2. They also range from moderate to high, except for the beauty ratings by the expertise Group R4, whose ICC was almost zero. This indicates that only the experts disagreed on which pictures are beautiful.

#### Creativity

**Figure 6:**
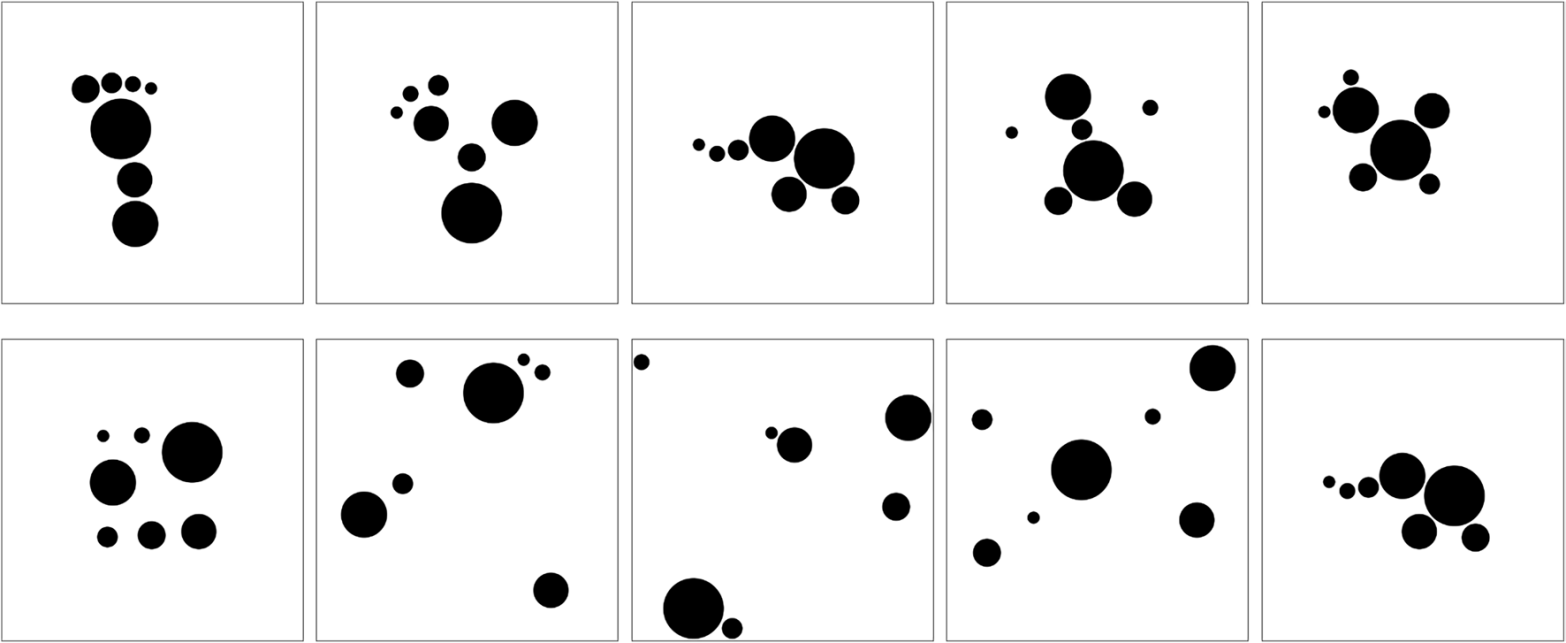
Upper row: The five best rated pictures in terms of creativity. Mean ratings are from left to right: 60.9, 49.5, 48.8, 46.2, and 45.3. Corresponding mean ratings for beauty are 52.1, 38.4, 47.4, 41.8, and 41.8. The expertise of the creators was C2, C3, C3, C4, and C1, respectively. All pictures are of semantic type. Lower row: The five best rated pictures in terms of creativity by rater Group R4. The mean creativity ratings by this group are 42.8, 42.3, 41.0, 40.6, and 40.1. Corresponding mean beauty ratings are 46.8., 39.4, 45.2, 37.7, and 36.1. The expertise of the creators was C4, C4, C4, C1, and C3, respectively. The picture types are: semantic, semantic, dispersed, dispersed, and semantic, respectively.

After ensuring that all expertise groups judged at least the creativity consistently, we further analyzed the data. The mean rating of creativity was 39.0 (*SD* = 5.61). The five winning pictures are shown in Fig. 6, which all allow semantic interpretations. This is quite different from the winning pictures of the beauty contest in Experiment 1B.

#### Effects of picture type and expertise

**Figure 7:**
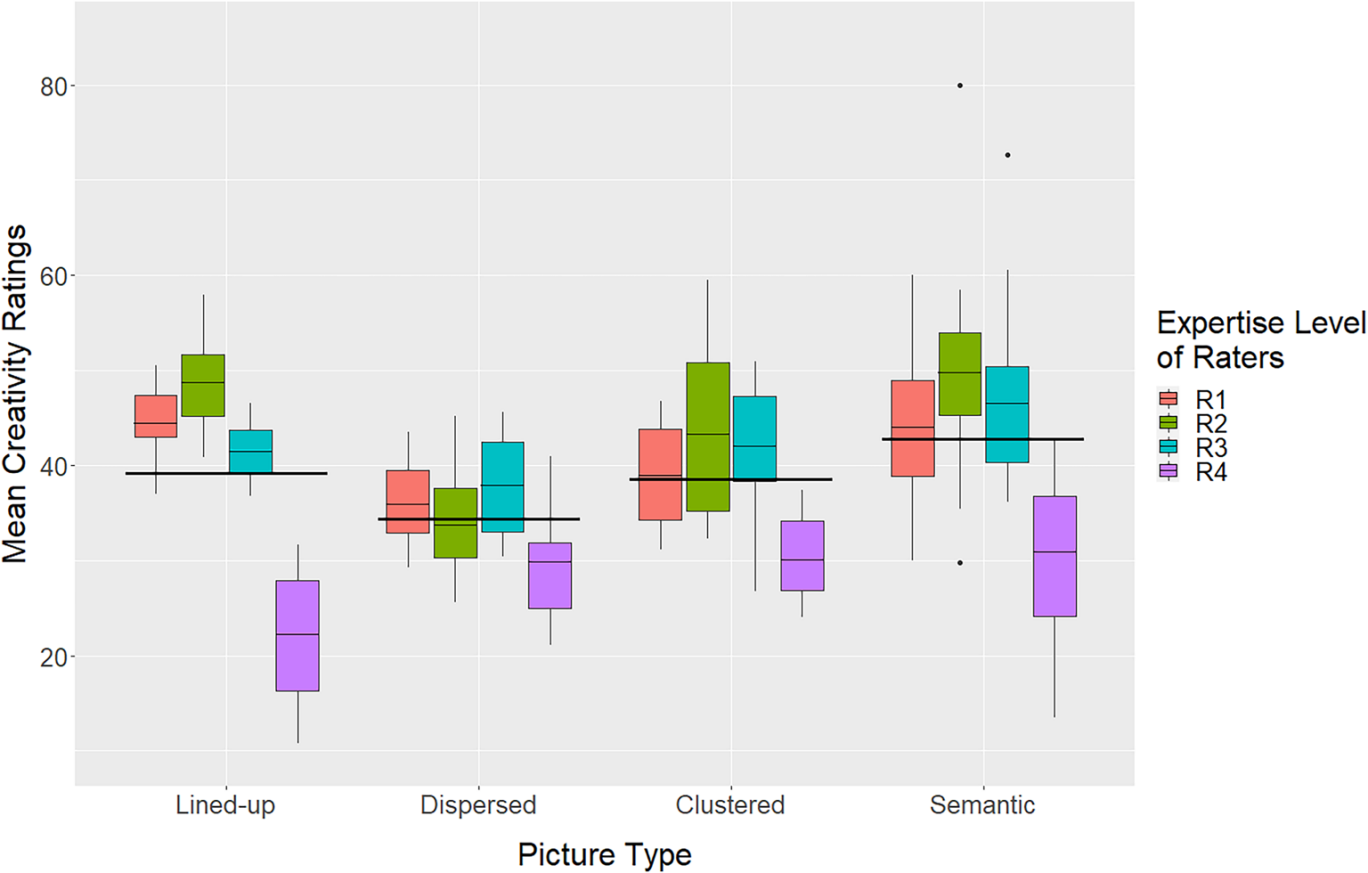
Mean creativity ratings for the picture types grouped by expertise level of raters.

How the creativity ratings by the four expertise groups varied across stimulus type is shown in Fig. 7. The data were subjected to a two-way ANOVA with the between-participants factor *expertise* (R1, R2, R3, and R4) and the within-participant factor *picture type* (lined-up, dispersed, clustered, and semantic). It revealed a significant main effect of *picture type*, *F*(3,219) = 14.2, *p* < 0.001, PES = 0.173 (lined-up: 39.2, dispersed: 34.4, clustered: 38.6, and semantic: 42.8). According to follow-up tests, the mean rating of the semantic type was significantly higher than those of the other types (at least *p* < 0.05 for all), whereas the mean rating of the dispersed type was significantly lower than those of the other types (at least *p* < 0.05 for all). This result is in line with the outcome of the contest and confirms that pictures with a semantic content were rated relatively high in terms of creativity.

In addition to *picture type*, the factor *expertise* also had a significant main effect, *F*(3, 219) = 56.7, *p* < 0.001, PES = 0.455. The mean values are R1: 41.1, R2: 44.1, R3: 42.2, and R4: 28.3. Follow-up tests revealed that the experts (R4) rated the pictures significantly lower (*p* < 0.001) than all other groups in terms of creativity, which also holds for the semantic category (*p* < 0.001). However, there was also a significant interaction between the two factors, *F*(9,219) = 4.79, *p* < 0.001, PES = 0.175, which was to be expected when looking at Fig. 7. Obviously, the ratings of Group R4 were somewhat unique. To analyze the role of R4 in more details, we computed an additional ANOVA without this group. It revealed that the factor *picture type* was still significant, *F*(3,164) = 19.8, *p* < 0.001. The ratings of the semantic pictures were again higher than those of the clustered ones (*p* = 0.006) and of the dispersed ones (*p* < 0.001), but not higher than those of the lined-up pictures (*p* = 0.589). The factor *expertise* was no longer significant, *F*(2,164) = 2.58, *p* = 0.079.

When we compared the ratings by Group R4 across picture types, we found that they differed significantly (*p* = 0.005). The ratings of the lined-up pictures were lower than those of the other types (*p* < 0.05 for all), while the ratings of the other three types did not differ to each other significantly. To get an impression of what the experts (R4) considered as creative, their five highest rated pictures are also shown in Fig. 6. As can be seen, although three of them are also of the semantic type, most of the pictures are more abstract than the winning pictures.

#### Predictive potential of the evaluators

We also examined how well the average creativity ratings of the pictures can predict the expertise of their creators. It was found that the Spearman correlation between ratings across all rater groups and creator expertise was not significant, *r*_*s*_ =  − 0.068, *p* = 0.317. However, when we considered the mean ratings by the individual rater groups, we found, as expected, that the ratings by experts (R4) were positively correlated with the expertise of the creators, *r*_*s*_ = 0.237, *p* = 0.041 (one sided). In contrast, the ratings by non-experts (R1, R2) were unexpectedly negatively correlated with creator expertise (R1: *r*_*s*_ =  − 0.353, *p* = 0.008; R2: *r*_*s*_ =  − 0.280, *p* = 0.038). The ratings by the quasi-experts (R3) did not correlate with the expertise of the creators, *r*_*s*_ = 0.166 (*p* = 0.226). These results reflect a difference in the evaluation criteria between experts and non-experts. It seems that only with the ratings by the experts (R4) it is possible, at least to some extent, to directly identify creative individuals. However, the prediction of creativity can be improved by combining the ratings by the experts (R4) and non-experts (R1) in a multiple regression. In this way, 21% of the variance can be explained, *F*(2,52) = 6.8, *p* = 0.002 (coefficients for R1 and R4 are − 0.057, *p* = 0.01, and 0.037, *p* = 0.047, respectively).

#### Beauty

As mentioned, the requirement to also evaluate the pictures in terms of beauty served to prevent participants from simply assessing beauty as a heuristic for evaluating creativity. However, the beauty ratings are also of interest in their own right, and furthermore in comparison to the creativity ratings and to the beauty ratings in Experiment 1. The mean beauty rating was 40.0 (*SD* = 7.07), which is similar to the mean beauty (42.6) in Experiment 1. The five pictures rated as most beautiful are shown in Fig. 9. As can be seen, the first three and the fifth are of the lined-up type, similar to Experiment 1.

#### Effects of picture type and expertise

**Figure 8:**
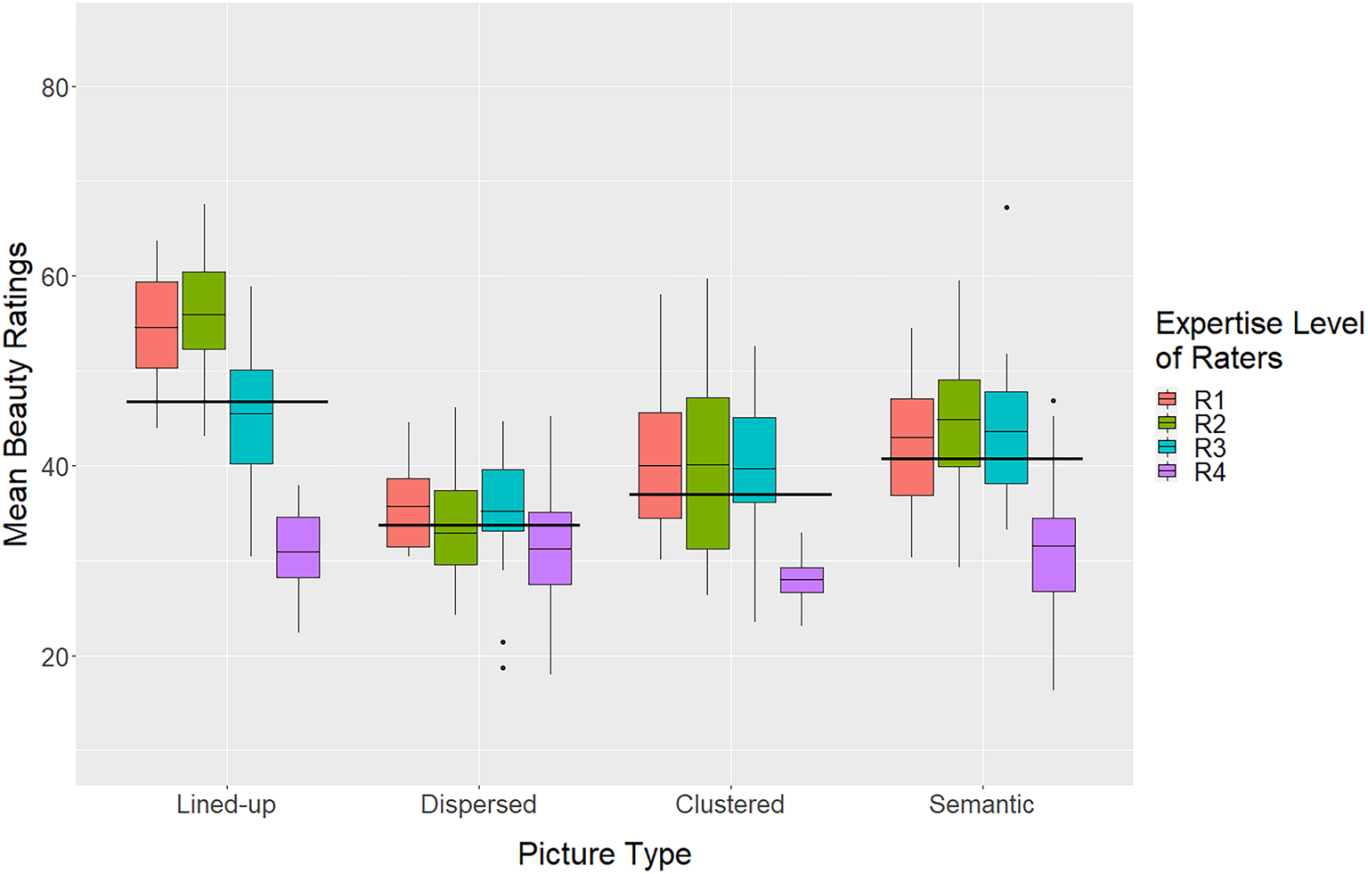
Mean beauty ratings for the picture types and different expertise levels of raters.

The beauty ratings were subjected to a two-way ANOVA analogous to the one used for the creativity ratings. It revealed a significant main effect of *picture type*, *F*(3,219) = 32.0, *p* < 0.001, PES = 0.320 (lined-up: 46.7, dispersed: 33.8, clustered: 37.0, and semantic: 40.8). As can be seen in Fig. 8, pictures with lined up elements were, on average, again liked more than those of all other types (*p* < 0.001). Furthermore, the mean beauty rating of semantic picture type was significantly higher than those of the dispersed type (*p* < 0.001) and the clustered type (*p* = 0.045).

The main effect of *expertise* was also significant, *F*(3,219) = 39.5, *p* < 0.001, PES = 0.368. As for creativity, the experts (R4) rated the pictures significantly lower than the other groups (*p* < 0.001). However, there was also a significant interaction between the two factors, *F*(9,219) = 5.05, *p* < 0.001, PES = 0.182. As can be seen in Fig. 8, the ratings by the experts (R4) did not differ substantially across picture type, whereas Groups R1 and R2 rated the lined-up pictures significantly higher than all other picture types (*p* < 0.01). Furthermore, Group R3 rated the lined-up pictures higher than the dispersed type pictures (*p* < 0.05), but not higher than the other two picture types.

**Figure 9:**
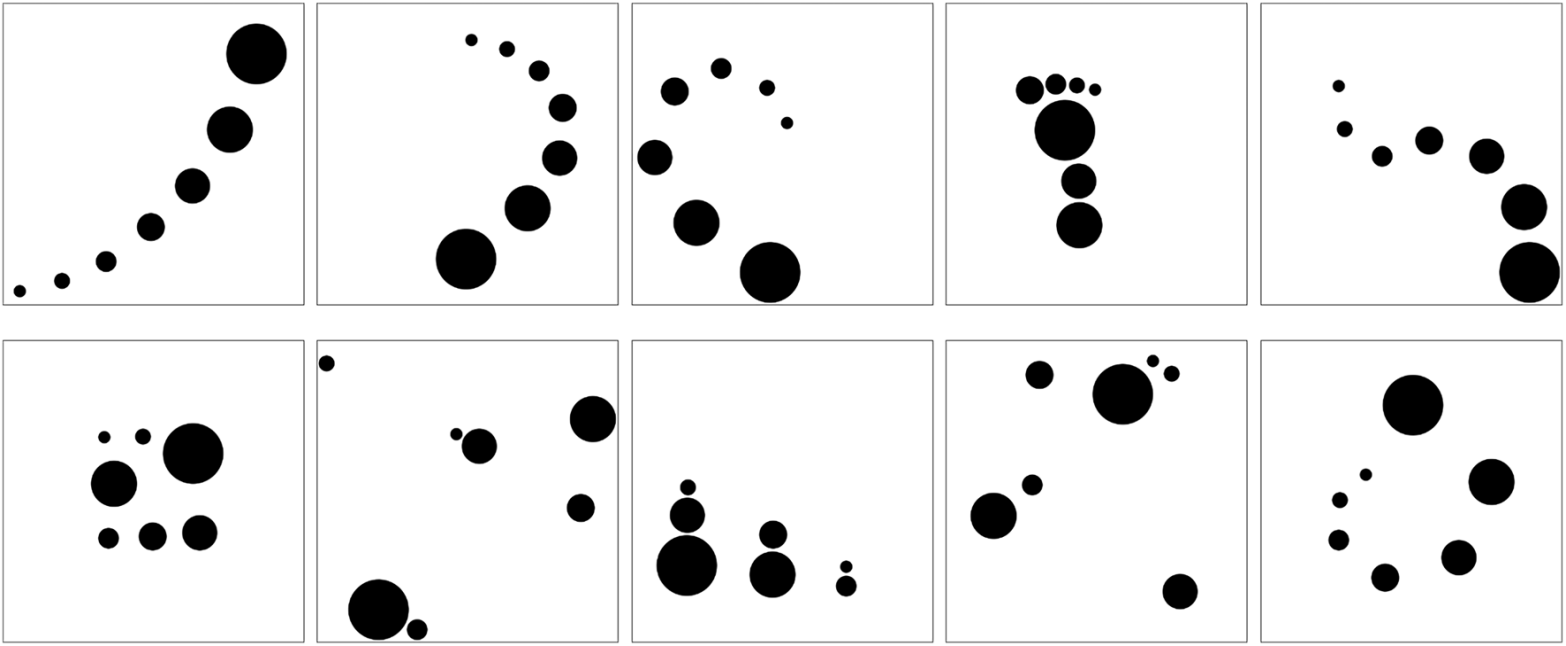
Top row: The five best rated pictures in terms of beauty. Mean ratings are from left to right 54.6, 53.1, 52.3, 52.1, and 51.5. Corresponding mean ratings for creativity are 32.8, 37.1, 37.9, 60.9, and 42.9. The expertise of the creators was: C2, C2, C2, C2, and C1. Bottom row: The five best rated pictures in terms of beauty by rater Group R4. The mean beauty ratings by this group are: 46.8, 45.2, 45.2, 39.4, and 38.0. The respective mean creativity ratings are: 42.8, 41.0, 33.7, 42.3, and 24.4. The corresponding picture types are: semantic, dispersed, semantic, semantic, and lined-up. The expertise of the creators was: C4, C4, C1, C4, and C1, respectively.

Overall, the present results are similar to those in Experiment 1. Pictures with lined up elements were liked most. However, in the present case, the experts (R4) performed quite differently. To get an idea of what they preferred, we also show in Fig. 9 the five pictures they rated highest. Three of them are of the semantic type. However, all are relatively abstract and complex, compared to the pictures generally rated as the most beautiful.

#### Predictive potential of the evaluators

To investigate the extent to which the beauty rating of an image depends on the expertise of its creator, we first computed an overall Spearman correlation, which was not significant,* r*_*s*_ =  − 0.098 (*p* = 0.148). In the next step, we also computed the correlation for each rater group. As a result, the mean ratings for group R1 correlated negatively with creator expertise (*r*_*s*_ =  − 0.286, *p* = 0.034). There was a similar tendency for group R2, which, however, failed to reach significance (*r*_*s*_ =  − 0.230, *p* = 0.091). For the experts (R3, R4) the correlations were positive, but not significant (R3: *r*_*s*_ = 0.076, *p* = 0.581; R4: *r*_*s*_ = 0.159, *p* = 0.247).

#### Relation between creativity and beauty

Finally, we examined how closely the ratings of creativity and beauty are related. Not only the two corresponding mean values are rather similar, but further analyses also revealed that the correlation between the two variables was positive and significant, *r* = 0.552 (*p* < 0.001). Nevertheless, the two ratings shared only about 31% of the variance. Therefore, we also computed the correlations within and between the different rater groups, which are shown in Table 3. Obviously, within groups they ranged from 0.51 (R4) to 0.77 (R2). However, what can also be seen is that the correlations between the experts’ (R4) creativity ratings and the non-experts’ (R1, R2) beauty ratings were significantly negative. Furthermore, the beauty ratings by the experts (R4) are not related to the creativity ratings by the non-experts (R1, R2) and the quasi-experts (R3).

**Table 3:** Correlations between the mean creativity ratings and the mean beauty ratings for the different rater groups.

		Creativity			
		R1	R2	R3	R4
Beauty	R1	0.635***	0.642***	0.270* (*p* = 0.046)	− 0.526***
	R2	0.607***	0.769***	0.295* (*p* = 0.029)	− 0.442***
	R3	0.436***	0.546***	0.624***	− 0.100 (*p* = 0.47)
	R4	− 0.225 (*p* = 0.099)	− 0.081 (*p* = 0.56)	0.092 (*p* = 0.50)	0.509***

*p < 0.05, ***p < 0.001.

### Discussion

In this second part, participants had to evaluate the pictures composed in the creativity contest of the first part in terms of creativity and beauty. If we compare the created pictures with those composed in the beauty contest in Experiment 1, it is striking that they are more evenly distributed across the different picture types. This resulted from the fact that the proportion of pictures with lined up elements was reduced compared to Experiment 1, while the proportion of semantic pictures was increased, suggesting that fewer typical pictures were composed to express one’s creativity than to create beauty. Instead, pictures with semantic content were relatively often used for this purpose. This interpretation of the picture type frequencies in the creativity contest is confirmed by the ratings in Part B. Pictures with semantic content were rated higher in creativity, and pictures of the lined-up type lower compared to the beauty ratings in Experiment 1. As a consequence, the assessment of the semantic pictures changed from being the worst in terms of beauty to the best in terms of creativity. This change indicates the application of different evaluation criteria in the two tasks.

However, there were also striking differences between the rater groups. First of all, the average ratings by the experts (R4) were generally lower than those of the other groups. Furthermore, they rated the lined-up pictures even worse than the other types. In contrast, the other groups rated the lined-up and semantic pictures similarly high. Despite these differences, not only the five winning pictures were of the semantic type, three of the five pictures rated highest on creativity by the experts (see Fig. 6) are also of the semantic type, even the two best.

Looking at the beauty ratings, they are similar to those in Experiment 1B, except that (1) the experts (R4) showed no preference for any picture type and thus no preference for the lined-up type, and (2) the other groups (R1, R2, R3) showed an increased aesthetic preference for the semantic type. It therefore appears that the additional creativity assessment task had some effect on the beauty ratings, at least for the experts. The creativity-assessment task presumably activated their controlled processes, which then also led to a more critical evaluation of beauty. It should also be noted that the ICC of the experts was not significant for the beauty ratings. The increased preference of the other groups for the semantic type could be a consequence of the increased number of semantic pictures, which also increased the probability of beautiful pictures.

Regarding the prediction of creators’ expertise, we found that only the creativity ratings by experts (R4) are positively and significantly related to it. In contrast, for non-experts (R1, R2), there was a negative correlation, which offers the possibility to also identify creative individuals through products that received a low evaluation by these groups. Indeed, by combining both experts’ and non-experts’ ratings in a multiple regression, the prediction of creativity could be improved. Interestingly, there was also a significant negative correlation between non-experts’ (R1, R2) *beauty* ratings and creators’ expertise.

Concerning the relation between creativity and beauty ratings, they were positively related for all rater groups. However, the creativity ratings by the experts (R4) were negatively correlated with the beauty ratings by the non-experts (R1, R2). This means that pictures assessed by experts as creative were rated as not beautiful by non-experts and those assessed by experts as not creative were rated as beautiful by non-experts. Finally, we found that beauty ratings by experts (R4) were unrelated to the creativity ratings by all other groups.

## General discussion

In this study, we examined the production and evaluation of visual compositions in terms of beauty and creativity, with special attention to the role of artistic and design expertise. Our basic procedure was similar to that of the Consensual Assessment Technique (CAT), often used in domain-specific creativity research^3^. Rather than evaluating a person's personality or mental processes as more or less creative, this technique relies on evaluating a person's products in this regard. Two experiments were conducted, each with a production part (A) and an evaluation part (B). Artistic and design expertise was assessed among both creators and evaluators using self-report.

In the first experiment, Part A was conducted as beauty contest, i.e., the participants were motivated to compose a most beautiful picture from a given set of circular elements. In order to structure the relatively large number of pictures produced, they were classified into four categories: lined-up, dispersed, clustered, and semantic. The largest category was lined-up compositions, of which 85% were subsequently classified as typical for the provided elements. This is much more than the 4% to 33% typical compositions in the other picture categories. Since typical pictures are usually very popular^42,43^, we expected that lined-up compositions would receive the highest beauty ratings in Part B of Experiment 1, which was indeed the case. Not only was this type the best rated, but all five winning pictures were also of the lined-up type. Moreover, the best ratings of lined-up pictures were given by raters with expertise levels R2 and R3. This can be interpreted in the sense that these raters already had some knowledge about beautiful design but were still grounded in everyday beauty^49,57^.

The smallest category was pictures with semantic content. Interestingly, although comprising more than half of the participants, neither of the two creator groups with the highest expertise (C3, C4) contributed a composition to the semantic category. Rather, many of the experts created lined-up pictures, while the others in this group composed more than half of the dispersed compositions. When we examined whether beauty ratings had the potential to predict creator expertise, we found no significant relationship between these two variables. This could mean that expertise is not necessarily involved in composing beautiful picture, at least with the simple means available here.

After exploring the basics of pictorial composition and the corresponding beauty assessment in our first experiment, important questions were: What would change if the task was to produce a creative picture instead of a beautiful one? Do experts have an advantage in this respect? Are experts better at evaluating creativity than non-experts?

These and related questions were investigated in our second experiment, whose first part was announced as creativity contest, i.e., the goal of the participants was to compose a most creative picture. Interestingly, it turned out that much less lined-up compositions were produced, but more semantic ones, compared to Experiment 1. Consequently, the produced pictures are more evenly distributed across picture categories. The experts (C4) did not produce a single lined-up picture under the creativity condition, but some of them have now composed semantic pictures. This suggests that the experts were well aware of the reciprocal relation between typicality and creativity, and therefore avoided composing typical pictures.

The creativity ratings in Part 2B revealed not only that this time all five winning compositions belonged to the semantic type, but also that, on average, the semantic compositions received the highest ratings. Thus, the average rating of semantic pictures changed from the worst in Experiment 1 in terms of beauty to the best in terms of creativity in Experiment 2. These striking differences clearly indicate the application of different evaluation criteria in the two tasks. In addition, there were also significant differences between the rater groups. First of all, experts (R4) rated the pictures less creative than the other groups, especially lined-up and semantic ones. This is in line with Runco, et al.^7^ who also found generally lower creativity ratings by experts. Nevertheless, the observed advantage of semantic pictures over lined-up ones was mainly due to Group R4, because the other groups rated these two types similarly high. Moreover, when we consider the five pictures rated the highest in term of creativity by Group R4 (Fig. 6), we find that, except two dispersed ones, they are also of the semantic type. However, some of them are more abstract than the winning ones. Thus, taken together, the results suggest that experts (R4) were motivated by their “need for cognitive enrichment”^26^ to process the compositions more cognitively and therefore considered a challenge in interpreting a composition as indication of high creativity. This interpretation is also in line with the idea that experts have a more elaborated mental structure with respect to beauty and creativity, and therefore need more challenges to arouse their curiosity^48,50^.

As mentioned, the creativity ratings in Experiment 2 differed largely from the beauty ratings in Experiment 1. Similar differences are also evident within Experiment 2, in which the pictures also had to be rated in terms of beauty. This is due to the fact that the beauty ratings in both experiments were rather similar. As in Experiment 1, compositions with lined up elements were again liked most. This time, however, the semantic pictures were not the worst, but more liked than the dispersed ones. One reason for the increase could simply be the increased number of semantic compositions, including the winning images, which were also well liked. The relatively high similarity of the beauty judgments between the two experiments despite their different context and participants demonstrates the high reliability of our results.

When we look more closely at the relation between creativity and beauty within Experiment 2, we find that their overall correlation (Table 3) is positive and significant, even within each rater group. This indicates that creativity and beauty are positively related. What persons find creative they also find beautiful, and vice versa, at least to a certain extent. However, this result does not imply that there was also generally high agreement between rater groups. Although the agreement was positive and significant for the beauty ratings in Experiment 1 (Table 1), this was no longer the case in Experiment 2 (Table 2). In our second experiment the beauty ratings by experts (R4) were unrelated to the ratings by the other rater groups. One reason could be that there was even no consistency within this group, which is in line with other results^50,53,54^.

The rater consistency was somewhat different for the creativity ratings. Not only was the intraclass correlation, although relatively small, also significant for group R4, there was additionally a positive correlation with the quasi-experts (R3). Thus, artistic expertise had a greater impact in Experiment 2 than in Experiment 1. The task of evaluating the creativity of the pictures was presumably motivating particularly for the experts to process them cognitively and to apply their specific knowledge^26^. This processing mode was then probably also active during the beauty ratings and led to novelty being weighted higher than typicality.

### Are experts needed for identifying creative individuals?

As mentioned in the Introduction, questions concerning the role of expertise is generally of great interest in creativity research^4,7,49,55^, and in particular in the context of CAT. A crucial question is whether experts are really needed for the evaluation of product creativity, which can also mean the identification of creative individuals. Some researchers have questioned whether this is the case^7,56^, while others^55^ maintain that judgments made by non-experts are invalid unless they agree with expert judgments.

In the present study, we attempted to find answers to this and related questions by examining the degree to which the observed ratings by the different rater groups are predictive for creator expertise, assuming that creators' self-assessed expertise also reflects, at least to some extent, their creativity. First of all, it can be said that beauty ratings are not well suited to identify creative individuals. In Experiment 1, there was no significant correlation between the beauty ratings of the pictures and the expertise of their creators, which was also the case in Experiment 2 for the quasi-experts (R3) and the experts (R4). There were significant correlations for the non-experts (R1, R2), which, however, were rather small and negative.

Importantly, the predictive power of creativity was substantial. We observed a positive correlation between the ratings by experts (R4) and the expertise of creators, indicating that this group was able to identify creative individuals, at least to some degree. A similar result was observed in the context of drawings^73^. To our surprise, though, there were also significant correlations for non-experts (R1, R2). However, they were again negative, suggesting that non-experts rated pictures composed by non-experts as creative and those created by experts as non-creative. Although this is a misclassification, it could nonetheless be used to improve expertise prediction because of its systematic nature. By adding the creativity ratings by the non-experts in a multiple regression, we were able to more than double the explained variance of the creators' expertise.

Even though the expertise required for a valid assessment of creativity might also depend on the product area^38,39^, the present results nevertheless support the conjecture that experts’ assessments of product creativity can be useful for identifying creative individuals, which is good news with regard to the validity of the CAT. According to the dual-process model^26^, the experts’ elaborated concepts of beauty, creativity, and their relation allows them to suppress direct emotional responses^10^ to pictures and process them more cognitively. This way, more novel and challenging pictures, often composed by experts, could be appreciated, and assessed as creative. In contrast, non-experts seem to have evaluated the images more directly and emotionally, and therefore not only preferred more simple pictures, but also assessed experts’ more novel and abstract compositions as non-creative. Due to their lack of artistic knowledge, presumably, they often used their impression of beauty as heuristic for assessing creativity. As we have seen, this led to opposite evaluation, which, however, could also be used for identifying creative individuals. Further studies need to show whether non-expert creativity assessments can always be applied in this way.

### Performance of the creators

So far, we have looked at the differences between the rater groups in great detail. But what can be concluded from the differences between the creators' expertise groups? In terms of beauty, the creators seemed to have largely agreed that lined up pictures were prototypical and therefore most appealing. The situation was quite different with respect to creativity. As mentioned earlier, lined-up compositions can also be considered as semantic pictures, albeit more prototypical and simple ones. Thus, if a creative product is understood as a novel deviation from prototype, then it seems that many non-experts tried to demonstrate their creativity by small deviations within the semantic category. Accordingly, they have created somewhat more complex semantic compositions, but which are nevertheless easily recognizable. Although experts avoided to create prototypical pictures, some of them also remained in the semantic category, although at a greater distance from the prototype, i.e., they composed more abstract and less easily recognizable pictures. Other experts, however, left the semantic category and produced rather abstract dispersed and clustered compositions.

In any case, our result that only the creativity ratings by experts (R4) were positively correlated with the creators' expertise level indicates that the strategies applied by the expert creators and the resulting pictures were appreciated by the expert raters. This agreement probably reflects the common concept of creativity between the experts (C4, R4).

### Limitations

Although the present study revealed various new and interesting results, it also has limitations. For example, because it is difficult to recruit experts for such experiments, the distribution of participants among the expert groups was uneven and varied from experiment to experiment, which is not optimal. Furthermore, in contrast to Experiment 1, students from the Royal College of Art in London participated in Experiment 2. It can be assumed that at least some of them had a higher expertise and creativity than the students from the HTWG in Konstanz. This difference might limit the comparability between the two experiments. However, the results that can be compared at all between the two experiments are quite similar, and the differences between the noncomparable results are plausible.

Another limitation is that we simply assumed that self-assessed expertise also reflected creativity, rather than measuring it. Although category four of our expertise scale was intended to identify active and creative persons (“I am an artist/designer or the like”), it might have been better to additionally require a separate self-assessment of artistic creativity, at least for the creators.

## Conclusions

The present study shows that expertise is not relevant when it comes to composing beautiful images from simple elements. Experts and non-experts were largely in agreement about what is beautiful. The situation is different when composing creative images. In this task, experts have a clear advantage in both production and evaluation. The experts were the only group that could identify creative individuals directly on the basis of the evaluation of product creativity.

## Data Availability

The datasets and pictures generated and/or analyzed during the current study are available in the OSF repository, 10.17605/OSF.IO/TXJFS.
